# Identifikation von lernfördernden Maßnahmen zur Einführung von digitalen und assistiven Technologien (DAT) in Prozesse der pflegerischen Versorgung: eine qualitative Studie

**DOI:** 10.1007/s16024-022-00372-4

**Published:** 2022-06-14

**Authors:** Lisa Geist, Ursula Immenschuh, Patrick Jahn, Denny Paulicke, Max Zilezinski, Christian Buhtz, Sebastian Hofstetter

**Affiliations:** 1 Katholische Hochschule Freiburg, Lehrstuhl für Berufspädagogik im Gesundheitswesen, Karlstr. 63, 79104 Freiburg i.Br., Deutschland; 2 Universitätsmedizin Halle (Saale), AG Versorgungsforschung | Pflege im Krankenhaus, Department für Innere Medizin, Medizinische Fakultät, Martin-Luther-Universität Halle-Wittenberg, Ernst-Grube-Str. 40, 06120 Halle (Saale), Deutschland; 3 Medizinische Fakultät, Dorothea Erxleben Lernzentrum Halle (DELH), Projekt FORMAT, Martin-Luther-Universität Halle-Wittenberg, Magdeburger Str. 12, 06112 Halle (Saale), Deutschland

**Keywords:** Pflegepädagogik, Pflegeprozessplanung, Assistive Technologie, Digitale Transformation, Pflegerobotik, Nursing education [H02.478.395.413], Nursing process [N04.590.233.508], Digital Transformation [L01.143.320.800], Assistive technology, Care robot

## Abstract

**Hintergrund:**

Digitale und assistive Technologien (DAT) finden Eingang in die Versorgung. Konzepte, Pflegende strukturiert an die neuen Technologien heranzuführen, fehlen. Daher macht dieser Aufsatz einen Vorschlag, Pflegefachpersonen im Umgang mit DAT anzuleiten, zu beraten und zu schulen.

**Ziel:**

Die Arbeit fragt, wie Pflegefachpersonen einen strukturierten Ansatz der Sensibilisierung, Qualifizierung und Erprobung hinsichtlich DAT erleben und bewerten. Verändert ein transformativer Lernansatz die Bereitschaft DAT einzusetzen?

**Methode:**

Pflegende wurden hinsichtlich DAT sensibilisiert. Pflegende wurden in der Anwendung von 2 Robotiksystemen und eines passiven Exoskeletts geschult, um diese anschließend zu erproben. Das Erleben der strukturellen Ausgestaltung des Edukationsansatzes wurde durch Interviews und durch die qualitative Inhaltsanalyse nach Kuckartz eingeschätzt.

**Ergebnisse:**

Alle 5 Pflegefachpersonen verfügen über eine 3‑jährige Ausbildung. Zwei nehmen neben pflegerischen auch Aufgaben der Wohnbereichsleitung wahr. Das Vorgehen wird durch alle Befragten positiv bewertet. Der strukturierte Ansatz erhöht das Interesse und die Bereitschaft DAT perspektivisch in den Pflegeprozess zu integrieren. Es zeigt sich, dass die Passgenauigkeit auf pflegerelevante Probleme und die Notwendigkeit DAT durch die Arbeitgeber verfügbar zu machen entscheidend sind, um DAT praktisch einzusetzen.

**Schlussfolgerung:**

Ein strukturiertes Konzept erhöht die Einsatzbereitschaft von DAT in der pflegerischen Versorgung. Einer mangelhaften Implementierung von DAT liegen u. a. fehlendes Wissen und fehlende Aus-, Fort- und Weiterbildungskonzepte zugrunde. Die angestoßene Reflexion ermöglicht es DAT auf spezifische Pflegeprobleme zu prüfen, spezielle Situationen im Versorgungsprozess zu berücksichtigen und Anwendungshürden abzubauen.

**Zusatzmaterial online:**

Zusätzliche Informationen sind in der Online-Version dieses Artikels (10.1007/s16024-022-00372-4) enthalten.

## Abstract (en)

**Background:**

Digital, assistive technologies (DAT) are finding their way into care processes. There are no concepts for introducing nursing professionals DAT in a structured manner. A structured concept makes sense for a sustainable implementation of DAT. This paper suggests a guideline to train nurses in dealing with DAT.

**Aim:**

The work addresses the question of how nursing professionals experience and evaluate a structured approach of sensitization, qualification and testing with respect to DAT. This is also intended to assess the extent to which a transformative learning approach changes the willingness of nurses to use DAT.

**Method:**

In a long-term inpatient facility, nurses have been made aware of DAT and instructed in its use. The nurses were trained in using two robotic systems and a passive exoskeleton. The experience and perception of the structural design of the educational approach were surveyed through interviews. Evaluation took place by qualitative content analysis according to Kuckartz.

**Results:**

All 5 nurses surveyed had completed 3 years of training in nursing care. 2 of the interviewees perform tasks in the management of the care unit. All interviewee rated the structured approach positively. The approach increases the interest to think about an integration of DAT. It turns out that the accuracy of fit of DAT to care-related problems as well as the necessity to make DAT available by employers are crucial prerequisites for DAT integration into practice.

**Conclusion:**

A structured concept can sustainably increase the willingness of nurses to use DAT. Poor implementation of DAT is based on a lack of knowledge and concepts for education and training. The reflection that has been initiated enables DAT to be checked for specific care problems.

## Einleitung

Der Einsatz von digitalen und assistiven Technologien (DAT) (z. B. robotische Systeme) wird als eine ergänzende Ressource in der Gesundheitsversorgung diskutiert (Hülsken-Giesler und Daxberger 2018). Unklar ist bis dato aber der Umfang, in dem assistive Technologien in der Praxis eingesetzt sind. Obwohl bereits einige DAT für die Anwendung in bestimmten Bereichen der Pflege verfügbar sind, kann von einer wirklichen Durchdringung oder sogar Entlastung im Pflegealltag nicht gesprochen werden. Das heißt, dass die entwickelten Systeme und Szenarien die Erlebniswelt und Bedürfnisse der Nutzer oftmals nicht ausreichend adressieren. Bisherige methodische Entwicklungsansätze sind häufig ungeeignet, die Geräte in eine breitere praktische Anwendung zu bringen (Buhtz et al. 2018; Paulicke et al. 2019). Hinsichtlich des Wissenserwerbs zum qualifizierten Einsatz von DAT in der Aus‑, Fort- und Weiterbildung der Gesundheits- und Pflegeberufe konnte keine Literatur gefunden werden.

Dieser Umstand wird beispielsweise mit einer fehlenden Integration der Thematik in die Lehrpläne der berufsbildenden Fachschulen begründet (Buhtz et al. 2020; Kehl 2018). Dadurch fehlt es bei den Pflegefachpersonen bezüglich DAT an theoretischem Wissen und praktischen Kompetenzen (Buhtz et al. 2020). Hasseler et al. (2020) zeigen am Beispiel der Versorgung von intensivpflichtigen Patienten und Patientinnen mit COVID-19 auf, dass die hochkomplexe Versorgung, unterstützt durch intensivmedizinische, technische Assistenzsysteme der Gerätemedizin, durch Pflegefachpersonal erfolgen muss. Das Personal benötigt für die Anwendung vermitteltes Fachwissen und spezifische Kompetenzen im Umgang mit den Assistenzsystemen.

In Anlehnung an die Überlegungen von Hasseler et al. ist zu konstatieren, dass eine zunehmende digitale Transformation deshalb auch neue Kompetenzen für das Pflegefachpersonal erforderlich macht (vgl. auch Kuhn et al. 2019). Dazu fordert der Deutsche Ethikrat (2020) ebenfalls eine curriculare Verankerung für die Aus‑, Fort- und Weiterbildung von Pflegefachpersonen, um Wissen und Kompetenzen zu DAT zu vermittelt. Diesen Bedarfen an Konzepten der Wissensvermittlung steht kaum ein passendes Angebot gegenüber (Paulicke et al. 2019). Da die Versorgung mit DAT als Teil der digitalen Transformation zu verstehen ist, gilt die Forderung nach Aneignungsmöglichkeiten für geschultes Fachwissen auch für eben diesen Bereich. Die in dieser Arbeit dargestellten Ausführungen zur behandelten Thematik beziehen sich ausschließlich auf die Sachlage in Deutschland.

## Zielsetzung und Fragestellung

Wie erleben und bewerten Pflegefachpersonen einen strukturierten Edukationsansatz, bestehend aus den Schritten „Sensibilisierung“, „Qualifizierung“ und „Erprobung“, hinsichtlich der Einführung von DAT in den Pflegeprozess? Durch die Beantwortung dieser Frage kann eingeschätzt werden, inwieweit sich die Bereitschaft von Pflegefachpersonen, DAT zu nutzen, verändert. Daher untersucht diese im Sommer 2020 durchgeführte Pilotstudie, wie Pflegefachpersonen einen strukturierten Edukationsansatz erleben und bewerten. Der Fokus liegt auf den Äußerungen der Pflegefachpersonen hinsichtlich der Tauglichkeit der einzelnen Schritte des strukturierten Edukationsansatzes.

## Methode

### Feldzugang und Auswahl der vorgestellten Technologie

Die Auswahl der DAT, also der konkreten technischen Artefakte, erfolgt in Orientierung an individuellen und spezifischen Problemlagen der Bewohner und Bewohnerinnen, wie sie sich aus der Pflegedokumentation und der Pflegeanamnese ergeben. Um ein patientenbezogenes Versorgungsproblem klarer zu beschreiben, wurde der Pflegebedürftigkeitsbegriff herangezogen (§ 14 SGB XI). Das „Neue Begutachtungsinstrument zur Feststellung von Pflegebedürftigkeit (NBA)“ beschreibt in 8 „Modulen“ (Wingenfeld und Schaeffer 2011; Wingenfeld et al. 2011) Pflegebedürftigkeit. Im konzeptionellen Rahmen dieser Arbeit sind diese „Module“ als „Bereiche der Pflegebedürftigkeit“ zu verstehen, innerhalb derer konkrete gesundheitliche Problemlagen identifiziert werden und eine dazu passende DAT zuzuordnen ist, um ein definiertes Pflegeziel zu erreichen. Zudem erfolgte die Auswahl der Technologien auf Grundlage der dazu bereits verfügbaren Evidenz, in der ein positiver Nutzen beschrieben ist (Kelly et al. 2021; Pu et al. 2020b, a; Zelik et al. 2022) sowie ihrer potenziellen Verfügbarkeit.

Ausgewählt und vorgestellt wurden 2 passive Exoskelette der Firmen „Laevo“ (Laevo, Rijswijk, Niederlande) und „Hunic SoftExo“ (Hunic GmbH, Baiersbronn, Deutschland), die Pflegefachpersonen durch das direkte Tragen am Körper bei unterschiedlichen Tätigkeiten körperlich entlasten. Das Exoskelett „Hunic“ wurde durch die Pflegefachpersonen für die Erprobung selbst ausgewählt. Aus dem Bereich der Kommunikationstechnologien wurden die Therapieroboter PARO (Fa. PARO Robots U.S., Inc.) und PLEO (Fa. Innvo Labs Cooperation, Hongkong und Nevada) ausgewählt. Ein ebenfalls verfügbares Telepräsenzsystem „Double 3“ (Fa. Double Robotics Inc., CA, USA) konnte aufgrund fehlender flächendeckender WLAN-Infrastrukturen und interner Restriktionen nicht für die Praxiserprobung berücksichtigt werden. Die Technologien waren den Autoren und Autorinnen zuvor bereits bekannt.

Durch bereits bestehende Kontakte im Projektteam konnte die langzeitstationäre Einrichtung als Praxispartner gewonnen werden. Aufgrund der Restriktionen im Zuge der COVID-19-Pandemie war der Einsatz der Technologien lediglich auf einem Wohnbereich möglich. In dem Wohnbereich der langzeitstationären Einrichtung leben 34 Bewohner und Bewohnerinnen. Das Pflegeteam des Wohnbereichs setzt sich aus Pflegehelfern und Pflegehelferinnen sowie 5 examinierten Pflegefachpersonen zusammen. Für die Intervention mit dem Edukationsansatz und die Interviews wurden die 5 examinierten Pflegefachpersonen des Wohnbereiches zur freiwilligen Teilnahme angefragt. Alle 5 Pflegefachpersonen signalisierten ihre Bereitschaft zur Teilnahme an der einen Zeitraum von 3 Wochen umfassenden Intervention im Zusammenhang mit der Einführung des strukturierten Edukationskonzeptes.

### Interview und Datenauswertung

Zur Datenerhebung wurde die Form des „informierten Interviews“ gewählt. Das zentrale Argument für eine informierte Gesprächsführung basiert auf dem Verständnis der Gesprächssituation als einem Kommunikationsprozess, bei dem die beiden Partner gemeinsam die Bedeutung von Fragen und Antworten konstruieren (Laudel und Gläser 2007). Zwar birgt das Vorgehen die Gefahr des „confirmation bias“, wenn Interviewerin und Durchführende der Schulung identische Person sind. Die Anwendung einer wissenschaftlich fundierten Interviewstrategie erhöht demnach sogar die Gefahr, selbstverständliche Annahmen nicht erkennen zu können, da die Interviewten Antworten als bekannt voraussetzen und diese daher nicht geben. Um die benötigten spezifischen und detaillierten Informationen einzuholen, müssen Forschende ihre Interessen jedoch in den Kontext ihrer Interviewpartner übersetzen, da sonst weder die Formulierung geeigneter Fragen noch das Verstehen des Gesprächspartners möglich wird (Laudel und Gläser 2007). Die Erhebung der Daten durch leitfadengestützte, informierte Interviews ermöglichte dann die Analyse auf Grundlage induktiv gebildeter Kategorien. Der methodische Rückgriff erschien im Zusammenhang mit dem Vorhaben als geeignet, da die Methode die Ermittlung relevanter, themenbezogener Informationen in einem angepassten und vertrauensvollen Gesprächsklima anhand vorgegebener Fragen und in der Folge dann aus dem Material heraus zulässt (Gläser und Laudel 2009). Dazu wurde auf Basis des Forschungsstandes zum transformativen Lernen und unter Berücksichtigung der einzelnen Konzeptschritte theoriegeleitet ein Interviewleitfaden entwickelt. Der Leitfaden (Zusatzmaterial online: Supplement 2) setzte sich aus den Abschnitten „allgemeine Schilderungen zu dem Einsatz von DAT“, „angewandte Methodenwahl und allgemeine Fragen zu den einzelnen Schritten des Schulungsansatzes“ sowie Vertiefungsfragen zu „Einsatzmöglichkeiten von DAT“ zusammen.

Die Interviews wurden aufgezeichnet, vollständig transkribiert und inhaltlich-strukturierend (Kuckartz und Rädicker 2018) systematisch analysiert. Damit war die Bildung von Kategorien in Anlehnung an die Prozessschritte nach Kuckartz und Rädicker (2018) induktiv möglich sowie in der Folge durch Gegenüberstellung in Matrizen beschreibbar. Die Ergebnisse der Untersuchung werden entsprechend dem COREQ-Statement berichtet (Tong et al. 2007). Das erste Interview erfolgte einen Tag nach der Erprobung, die weiteren Interviews dann 4, 5 und 6 Tage später. Sowohl die Umsetzung des Edukationsansatzes als auch die daran anschließenden Interviews wurden durch dieselbe Person durchgeführt.

### Intervention – strukturierter Ansatz der Sensibilisierung, Qualifizierung und Erprobung

**Figure 1:**
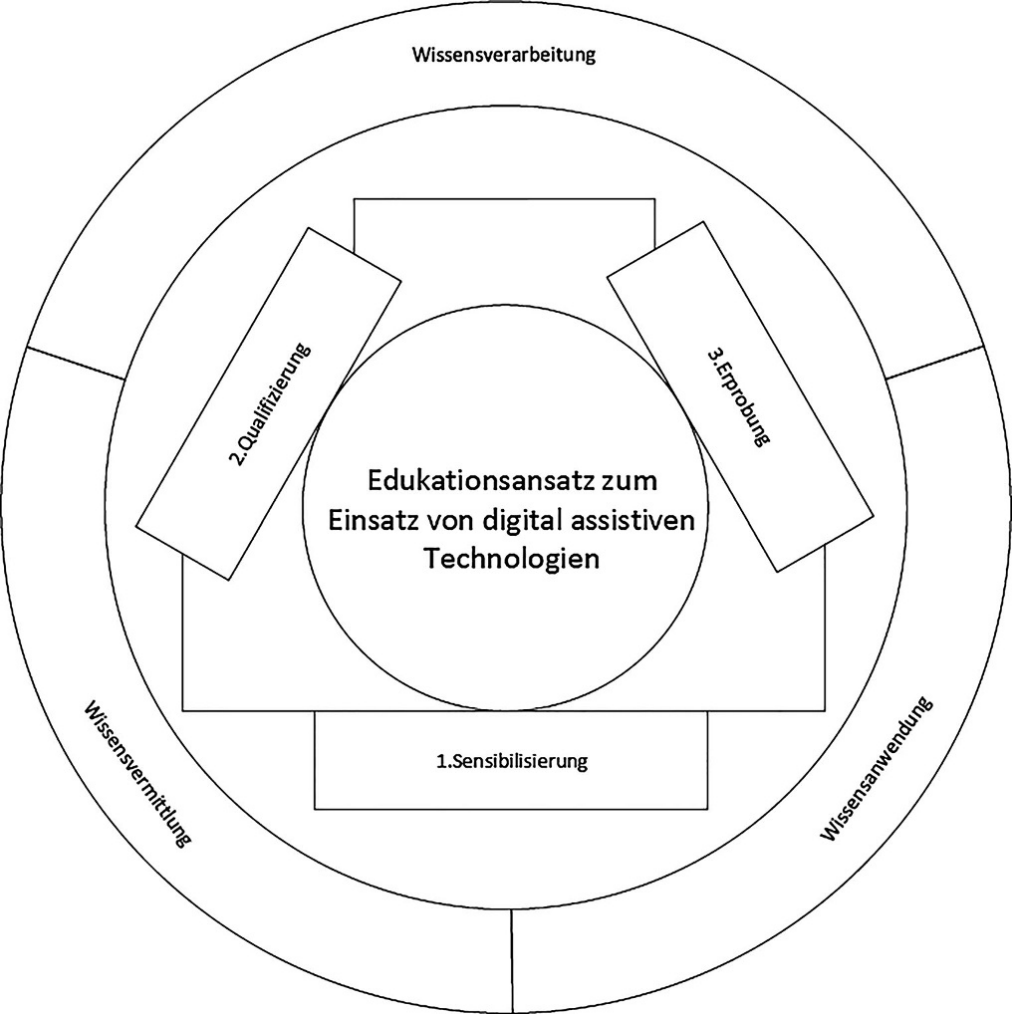

Ein Überblick über den strukturierten Edukationsansatz ist in Abb. 1 ersichtlich. Der strukturierte Edukationsansatz basiert auf den theoretischen Überlegungen zum transformativen Lernen nach Mezirow (Mezirow 1978, 1997, 2009). Ausgehend von den Ideen des lebenslangen Lernens (Hanft 2013) ermöglicht „transformatives Lernen“ durch eine reflexive Neubewertung erfahrungsbasierter Annahmen und Einstellungen die Neuinterpretation früherer Erfahrungen (Mezirow 1997). Dabei steht die Herausbildung neuer Bedeutungsperspektive durch eine „Dynamik des Lernens“ (intentionales, intuitives Lernen), eingebettet in einen Problemlösungsprozess, im Fokus; (Mezirow 1978, 1997, 2009; Singer-Brodowski 2011; Paulicke 2021). Durch den somit angestoßenen Prozess der „Transformation“, also der Ausprägung neuer Bedeutungsperspektiven hinsichtlich bis dahin als unumstößlich geltender Sinnzuschreibungen (Singer-Brodowski 2011; Paulicke 2021), wird es möglich, die eigene Erfahrung sowohl als Ausgangs- als auch Endpunkt für Möglichkeiten der geplanten Technikintegration in den Pflegeprozess zu denken und zu reflektieren. Aus diesem theoretischen Ansatz leitete sich in der Folge das dreistufige Vorgehen des Edukationskonzeptes ab.

In dem ersten Schritt der Sensibilisierung (in der ersten Woche) wurde in Einzel- und Gruppengesprächen, während der Arbeitstätigkeiten und zu den Übergabezeiten, Wissen zu bereits entwickelten Technologien sowie zu aktuellen Einsatzfeldern von DAT in unterschiedlichen Pflegebereichen vermittelt. Technik wurde dabei auch als eine durch die Pflege bereits in vielfältiger Weise genutzte Assistenz beschrieben (z. B. Blutdruckmessgeräte oder Pulsuhr), um den Transformationsprozess bei den Pflegefachpersonen damit anzustoßen und auf bereits gemachte Technikerfahrungen, im Sinne der Theorie Mezirows, zu rekurrieren. Anschließend fand eine allgemeine Vorstellung bereits verfügbarer DAT statt.

Im zweiten Schritt der Qualifizierung wurde das Pflegefachpersonal an den mitgebrachten Technologien geschult und in der Anwendung angeleitet, sodass in der zweiten und dritten Woche letztendlich 3 DAT (PARO, PLEO, Exoskelett Hunic) durch sie selbstständig angewendet werden konnten. Die Wissensvermittlung erfolgte anhand einer 2‑stündigen theoretischen Einführung in das Themengebiet, der Vermittlung von Wissen zu einer möglichen Integration in den Pflegeprozess und durch punktuelle Unterstützungsangebote während der Erprobung, wodurch die Handhabungskompetenz intensiviert wurde. Inhaltlich vermittelt die 90-minütige Sensibilisierungseinheit allgemeines Wissen zu DAT sowie spezifisches Wissen (Funktion, Anwendungsbereiche). Ziel ist es, Gesundheitsfachpersonen an DAT als zusätzliche Ressource für den Pflegeprozess heranzuführen. Dazu wurde auch diskutiert, wie anhand eines identifizierten Bedarfs DAT im Zusammenhang mit der Pflegeprozessplanung als ergänzende Ressourcen eingeplant werden können. Im Kontext der Pflegeprozessplanung werden die DAT als zusätzliche Ressource für das Erreichen der geplanten individuellen Pflegeziele der Bewohner und Bewohnerinnen verstanden. Im Rahmen einer Pflegevisite in der zweiten Woche erarbeitete das Pflegefachpersonal mögliche Szenarien, in denen DAT sinnvoll einsetzbar sind.

Im dritten Schritt erfolgte in der zweiten und dritten Woche eine Erprobung der DAT. Hierzu wurden die erarbeiteten Szenarien im Pflegealltag umgesetzt. Direkt im Anschluss an die Erprobung erfolgte eine einstündige Reflexion über die Wahrnehmung des Edukationsansatzes während der Erprobungsphase gemeinsam mit dem Pflegefachpersonal. Diese Reflexion wurde als Nachbesprechung innerhalb des Teams während der Arbeitszeit umgesetzt.

## Ergebnisse

### Teilnehmerinnen und Einrichtung

Da es sich um eine Pilotstudie handelt, wurde eine vergleichsweise kleine Anzahl von Teilnehmerinnen eingeschlossen. Da die Beschreibung des Erlebens und der Wahrnehmung des strukturierten Konzepts durch die Pflegefachpersonen vordergründiges Erkenntnisinteresse war und weniger die Verallgemeinerbarkeit der Ergebnisse, ist die Anzahl von lediglich 5 Probandinnen im Rahmen dieser Pilotstudie angemessen. Das Alter der Pflegefachpersonen in Jahren erstreckt sich von Ende 20 bis Mitte 40. Alle Befragten sind weiblich. Die Befragten weisen eine Berufserfahrung von 9 bis 18 Jahren auf. Zwei der Befragten sind gleichzeitig in der Leitungsebene der Einrichtung tätig.

### Wissens- und Kompetenzvermittlung

Als hilfreich im ersten Prozessschritt der Sensibilisierung wurden die Möglichkeiten nachzufragen und die Kommunikation auf Augenhöhe genannt:* „[…] man kommuniziert ja auf einer ganz anderen Ebene, wie wenn da jetzt jemand kommt, der […] drei Stunden Vortrag vorrattert“ (092006, 554* *ff.)*. Die enge Zusammenarbeit mit der Mentorin wurde als wichtige Kommunikationsbasis erachtet: *„Dieses Nachfragen […] war eigentlich gut. […] das Miteinander […] war sehr gut“ (092001, 180* *f.).* Die Kompetenzvermittlung im zweiten Schritt zur Nutzung der DAT während der Qualifizierung wurden als förderlich beschrieben. Die Wissensvermittlung wurde durch punktuelle Unterstützungsangebote während der Erprobung intensiviert. Durch die Praxiserprobung als dritter Schritt konnten eigene Erfahrungen gesammelt werden. Die Vermittlung des theoretischen Hintergrundwissen wird als Chance verstanden, eigene Ideen für individuelle Einsatzszenarien zu entwickeln: *„[…] Durch die Präsentation konnte man sich schon ein paar Ideen sammeln […] ein paar Ideen, wo man sagt, […] das könnte was werden, das könnte klappen“ (092002, 148* *ff.)*. Insgesamt wurde die methodische und strukturierte Umsetzung des Ansatzes durch das Gesundheitsfachpersonal als Mehrwert beschrieben, da die sensibilisierende Hinführung an den Bereich DAT einen sinnvollen und breiten Überblick über bereits verfügbare DAT und deren potenzielle Einsatzmöglichkeiten vermittelt: *„[…] also, das fand ich sehr gut, weil das hat […] ein bisschen Einblick gegeben, was ist der Sinn, was ist das Ziel dahinter, […] wofür können wir das nutzen“ (092005, 117* *ff.)*.

### Zeit

Das Pflegefachpersonal wurde durch die Sensibilisierung dazu angeregt, über den Faktor Zeit als Ressource zu reflektieren. Darüber hinaus greift das Pflegefachpersonal das Thema Zeit im Verlauf mehrfach auf. So wurde überlegt, wo durch den Einsatz von DAT Zeit eingespart werden könnte, und wie diese Zeitersparnis sinnvoll für die Bewohner und Bewohnerinnen zu reinvestierten ist. In zwei Aussagen wurde der Einsatz des Telepräsenzsystems, welches in den Schritten „Sensibilisierung“ und „Qualifizierung“ vorgestellt wurde, im Nachtdienst genannt: *„Wenn man einen Notfall hat, […] dann hat es eine halbe Stunde geklingelt, weiß ich, was in dem Zimmer ist? Ich kann da drüben nicht weg. Ja, für sowas wäre das richtig gut“ (092006, 218* *ff.); „Wenn ich mir jetzt vorstelle im Nachtdienst, die würden [das Telepräsenzsystem] uns was aufrufen, wenn ich auf dem anderen Wohnbereich bin und auf dem zweiten Wohnbereich klingelt es […] und ich sehe das, […] dass wär auf jeden Fall eine Riesenhilfe“ (092002, 145* *ff.).* Andererseits ist die Ressource „Zeit“ in der Gesundheitsversorgung knapp bemessen und entsprechend zu planen. So reicht die vorhandene Zeit nur für die bereits durchgeführten pflegerischen Tätigkeiten, wie beispielweise die Unterstützung oder Übernahme der Körperpflege, Essen und Trinken, Verabreichung von Medikamenten. Das Gesundheitsfachpersonal gibt im Bezug zum Zeitmangel an, für die Nutzung von DAT, v. a. der Kommunikations- und Therapierobotik, keine zeitlichen Ressourcen zu haben: *„Hätten wir diese Geräte, hätten wir persönlich keine Zeit dafür“ (092006, 192* *f.)*.

### Aspekte der Interprofessionalität

Das Pflegefachpersonal beschreibt es als wichtig, die Schulungsangebote weiteren Berufsgruppen zugänglich zu machen. Dieser Aspekt der Interprofessionalität bezieht sich auf alle Phasen des Edukationsansatzes: *„Wenn wir […] eine zusätzliche Betreuungskraft haben, die […] geschult ist und sagt, dass sie das macht, […] kann sie sich was anderes einfallen lassen […] verschiedene Möglichkeiten […], dann könnte das wunderbar funktionieren“ (092006, 194* *ff.)*. Bisher verfügbare Angebote für die Bewohner und Bewohnerinnen können durch die Technologien somit attraktiver gestaltet werden: *„Die zusätzlichen Betreuungskräfte hätten mehr Beschäftigungsangebot. […] Das ist zurzeit sehr mager“ (092001, 50* *f.)*. Durch die strukturellen Gegebenheiten sollen andere Berufsgruppen spezifisch und intensiv für den Umgang geschult werden, sodass eigene Ideen entwickelt werden können: *„Mitarbeiter, […] schon in dem Sinne geschult werden, wie funktioniert das Ganze, und inwieweit kann ich das in verschiedenen Möglichkeiten anwenden“ (092006, 333* *ff.)*.

### Passgenauigkeit

Es zeigt sich, dass die Passgenauigkeit der DAT auf pflegerelevante Probleme und die Notwendigkeit Technologien durch Arbeitgeber und Arbeitgeberin verfügbar zu machen entscheidende Voraussetzungen sind, damit Gesundheitsfachpersonen diese in der Praxis einsetzen. Die Pflegevisite als Hilfsmittel wird als wichtig beschrieben: *„Es war sehr hilfreich […] zu schauen, was gut in die Pflege passt, oder wo es noch Aspekte gibt, die gut überlegt werden müssen […]“ (092001, 3* *ff.)*.

Das Vorgehen zur Identifizierung der Passgenauigkeit wurde als sinnvoll wahrgenommen: *„[…] So bist du gekommen, […] hast erstmal geschaut, wo kann ich das machen; mit uns im Kontakt gewesen […], ist das geeignet, […] wo ist es nicht so günstig […]“ (092005, 182* *ff.)*.

Das im Verlauf des Edukationskonzeptes erworbene Wissen wurde als Mehrwert für die Identifizierung der Passgenauigkeit erkannt: *„[…] man hat erstmal gesehen […], was gibt es überhaupt alles, womit können wir uns beschäftigen […]“ (092002, 143* *f.)*. Dieser Mehrwert zeigt sich dann, wenn die Passgenauigkeit der DAT bezogen auf ein konkretes Pflegeproblem reflektiert wird und die Anwendung im Rahmen der Pflegeprozessplanung aus der Pflegeanamnese heraus umgesetzt ist.

### Informationsvermittlung

Als Mehrwert wird die Möglichkeit, überhaupt grundlegende Informationen zu DAT in der Pflege zu erhalten, dargestellt. Es wird eine Informationslücke hinsichtlich der Anwendung von DAT identifiziert: *„Ich kannte das [die DAT] nicht, woher auch, also weder von den Nachrichten noch irgendwo anders hab ich das gesehen“ (092006, 277* *f.).* Weiterhin wird eine Informationslücke dahingehend beschrieben, dass das medial gezeichnete Bild über vermeintliche Potenziale von DAT zu weit von einer pflegerischen Praxis entfernt ist und sich Pflegefachpersonal daher von der Thematik nicht angesprochen fühlen: *„Klar kennt man das aus der Presse, aber das ist ja immer so weit weg“ (092005, 303* *f.).*

### Schulungsbereitschaft und Akzeptanz

Die Schulungsbereitschaft des Pflegefachpersonals im aktuellen wie im zukünftigen Zeitraum ist hoch. Das eigene Team sowie die langzeitstationäre Einrichtung werden hierbei als sehr aufgeschlossen wahrgenommen: *„[…] wir sind da echt offen, also auch mein Team habe ich so erlebt, die sind ja wirklich auch nicht abgeneigt“ (092005, 134* *f.).* Es wird beschrieben, dass eine kontinuierliche Form der Weiterbildung in dem Bereich als sinnvoll angesehen wird. Das erworbene Wissen soll erhalten bleiben. Auch für weitere Thematiken wird die angewandte Vorgehensweise gewünscht: *„[…] dass das eben […] im Gedächtnis bleibt […], es ist auch für die Pflege wichtig […], das man vielleicht in den Genuss kommt, […] andere Sachen zu probieren“ (092005, 267* *ff.).*

### Didaktisches Vorgehen

Eine strukturierte und hinführende didaktische Aufbereitung, die mit einem theoretischen Einstieg beginnt und anschließend in einer praktischen Erprobung resultiert, wird als sinnvoll beschrieben: *„Zuerst so das Theoretische ein bisschen und hintendran […] das Praktische, das wäre schon ganz gut denke ich“ (092001, 234* *f.).* Die 3‑stufige Vorgehensweise, die ausgehend von der Sensibilisierung durch Vermittlung theoretischer Hintergründe einen Praxisbezug herstellt, wird als notwendig beschrieben, da somit die Neugier im eigenen Arbeitsfeld geweckt werden konnte: *„Ja, aber ich glaube, so wie Sie es gemacht haben, war’s schon gut, weil da ist die Neugier erstmal gekommen“ (092002, 164* *ff.).* Wichtig ist es, das Gesundheitsfachpersonal miteinzubeziehen. Ein weiterer Punkt ist daher die verständliche Aufbereitung des zu vermittelnden Inhaltes. Es wird eine Unterweisung durch neutrale Dritte, die von außerhalb der eigenen Institution kommen, als wichtig empfunden: *„Also das ist, wenn dann nicht jemand von außen kommt, […] schwer“ (092005, 221* *f.)*. Bezogen auf die durchgeführte Schulung wurde das strukturierte Vorgehen als eine geeignete Methode der Wissensvermittlung für die pflegebezogene Mensch-Technik-Interaktion beschrieben: *„[…] was gemacht wurde, das war schon echt top, da großes Lob auch nochmal“ (092001, 176* *f.).*

Die Befragten berichteten, dass die Umsetzung des 3‑stufig strukturierten Edukationskonzeptes dazu beiträgt Unsicherheiten abzubauen.

## Diskussion

Im Rahmen dieser Pilotstudie wurden Gesundheitsfachpersonen eines Wohnbereiches einer langzeitstationären Einrichtung in Interviews zu ihren Eindrücken hinsichtlich eines Edukationsansatz zur Anwendung von DAT befragt. Als theoretischer Rahmen zur Erarbeitung diente die Theorie des transformativen Lernens (Mezirow 1978, 1997).

Ziel war es zu untersuchen, inwieweit die schrittweise, strukturierte Heranführung an DAT Pflegefachpersonal den Praxiseinsatz erleichtert und ihnen die Reflexion ermöglicht, bereits vorhandene Kompetenzen auf den Einsatz von DAT zu übertragen sowie den Einsatz ihrerseits zu reflektieren.

Die theoretische Wissens- und Kompetenzvermittlung sowie die Integration des Pflegefachpersonals in den Prozess der aufeinander aufbauenden Schulungseinheiten wurden als ansprechend und gut empfunden. Durch das interaktive Vorgehen konnten eigene Ideen für Einsatzszenarien entwickelt werden. Somit können Gesundheitsfachpersonen nach dem Edukationsansatz aus eigenen Wissensbeständen heraus Ideen entwickeln, die sie als langfristig und nachhaltig für die Praxis sehen und diese perspektivisch als Multiplikatoren und Multiplikatorinnen an Kollegen und Kolleginnen weitergeben. Der Prozess des Überdenkens bereits verfestigter Vorannahmen, wie es das Konzept des „transformativen Lernens“ anregt, ermöglicht ein Verständnis für alternative Handlungsoptionen und Lösungsansätze. Dazu gehören z. B. Implementierungswissen, die Bewusstwerdung neuer Technologien als zusätzliche pflegerische Handlungsoption sowie die Umsetzung dieses Wissens in einer pflegerischen Praxis. Es ist aktuell davon auszugehen, dass durch den Umgang mit DAT eine Weiterentwicklung der eigenen Fachexpertise stattfindet und dieses neue Wissen dann auf die Technik übertragen und angewandt wird (Buhtz et al. 2020; Haucke et al. 2020). Die Aussagen der Pflegefachpersonen lassen darauf schließen, dass das 3‑stufige Vorgehen geeignet ist zu sensibilisieren, anhand von Anleitungen zu qualifizieren und eine Praxiserprobung zu ermöglichen. Aus den vorliegenden Daten kann abgeleitet werden, dass durch die Wissensvermittlung auch Kompetenzen erworben wurden. Somit kann der notwendigen Anforderung an den Erwerb neuer Kompetenzen im Zusammenhang mit der vielfach diskutierten digitalen Transformation entsprochen werden (Kuhn et al. 2019).

Die Kompetenzvermittlung durch den Edukationsansatz wurde von den Pflegefachpersonen als förderlich empfunden. Das deutet darauf hin, dass sich Wissen angeeignet, das Pflegefachpersonal Sicherheit erlangt und sich die Betrachtungsweise hinsichtlich des Einsatzes von DAT durch die Neuinterpretation als zusätzliche Ressource im Zusammenhang mit der Pflegeprozessplanung veränderte. Die Tatsache, dass diese Schulung den Erstkontakt des Gesundheitsfachpersonals mit derartigen Technologien ermöglicht hat, veranschaulicht, dass in der Versorgungspraxis bisher kaum DAT angekommen sind (Buhtz et al. 2018; Paulicke et al. 2019).

Die befragten Pflegefachpersonen regten auch Fragen dahingehend an, welche Tätigkeiten aus ihrer Perspektive besser an andere Berufsgruppen wie Ergo- oder Physiotherapeuten- und Therapeutinnen zu delegieren sind oder aus der Gruppe der Therapieberufe begleitet werden sollten. Durch die Übernahme von Aufgaben durch Ergo- oder Beschäftigungstherapeuten- und Therapeutinnen unter Zuhilfenahme von DAT wäre es möglich, das Wohlbefinden der Bewohner und Bewohnerinnen positiv zu beeinflussen. Deshalb ist es nach Ansicht der befragten Pflegefachpersonen wichtig, auch andere Berufsgruppen im unterstützenden Einsatz von ausgewählten DAT zu unterweisen. Das bedeutet, dass Pflegefachpersonen beginnen, DAT auch hinsichtlich Einsatzmöglichkeiten im Sinne eines multiprofessionellen, interdisziplinären Teams zu reflektieren. Gerade in diesem Punkt erfolgte eine reflexive Neuinterpretation der erprobten DAT im Sinne der Theorie Mezirovs. Der daraus erwachsende zeitliche Vorteil wird von den interviewten Pflegefachpersonen positiv beschrieben. Durch die Delegation bestimmter durch DAT unterstützter Tätigkeiten an andere Berufsgruppen wird die potenzielle Mehrarbeit, die durch den Einsatz von DAT entsteht, und damit einhergehende Verantwortung umgelagert. Das bedeutet, dass Pflegefachpersonen aktuell verfügbare DAT, die hauptsächlich im Bereich der Kommunikation und emotionalen Begleitung eingesetzt werden, eher als unterstützend für Therapieberufe (z. B. Alltagsgestalter, Ergotherapie) wahrnehmen. Für die eigene Berufsgruppe der Pflegefachpersonen und zur Unterstützung im Bereich der fachpflegerischen Tätigkeit (Grundpflege, Behandlungspflege) sowie ihrem Einsatz in Zusammenhang mit der Ablauf- und Pflegeprozessplanung sind aktuell verfügbare DAT noch überwiegend ungeeignet. Ein häufig aufgeführter Problempunkt, der Punkt der mangelhaften Passgenauigkeit digitaler und technischer Assistenzen auf pflegerelevante Problemfelder (Hergesell et al. 2019; Hofstetter 2019; Hülsken-Giesler 2019; Klebbe und Eicher 2019), wird durch die Pflegefachpersonen ganz deutlich benannt.

Damit sprechen die Pflegefachpersonen ein Thema von besonderer Relevanz an. Elementar für ihren Einsatz ist die Passgenauigkeit der DAT als eine ergänzende Maßnahme auf eine Versorgungsherausforderung. Die Passgenauigkeit von DAT im Pflegealltag kann im Rahmen der Pflegevisite identifiziert werden. Die Pflegefachpersonen identifizieren in ihrem Alltag ein Pflegeproblem, stellen eine Diagnose und definieren Pflegeziele auf Grundlagen ihrer Fachexpertise. Daraufhin legen sie Maßnahmen fest und planen die benötigten Ressourcen, die zur Verbesserung oder Lösung oder zumindest der Stabilisierung des Pflegeproblems beitragen. Daher ist eine Bestimmung der Auswahl der DAT und deren Einsatzmöglichkeiten durch die Pflegefachpersonen sinnvoll.

Das Pflegefachpersonal beschreibt das strukturierte Vorgehen des Schulungsansatzes aus seiner Sicht als sinnvoll, um Informationen über DAT in einem pflegerischen Kontext zu erhalten. Pflegefachpersonal ist dazu angehalten, orientiert am jeweils aktuellen Stand der Wissenschaft zu pflegen. Dazu muss jedoch das Wissen vorhanden sein, wie ein Zugang zu diesen Informationen möglich ist. Eine einmalige Schulung ist dafür nicht nachhaltig genug. Auf langfristige Sicht ist daher eine curriculare Verankerung in Form von Aus‑, Fort- und Weiterbildungsangeboten notwendig (Deutscher Ethikrat 2020). Dem entspricht auch die Aussage der Interviewpartnerinnen, die Wissensdefizite über bereits verfügbare und einsetzbare DAT als problematisch empfinden. In diesem Sinne wurde gerade der erste Schritt der Sensibilisierung als wertvoll beschrieben, da damit Wissenslücken geschlossen werden. Weiterhin wird der Mangel an verfügbaren Informationen zu einem regelrechten Einsatz von DAT geschildert. Dies deckt sich mit weiteren Forschungsergebnissen (Paulicke et al. 2019). Aktuell wirkt sich dieses Wissensdefizit hinsichtlich eines nachhaltigen, langfristigen Einsatzes von DAT negativ aus, da somit der Aufbau eines informierten Wissensstandes über einen schnelllebigen Technologiemarkt kaum möglich ist.

Ihre Bereitschaft, sich notwendiges Wissen zu DAT z. B. in Form von Fort- und Weiterbildungen anzueignen, beschreiben die Pflegefachpersonen als hoch. Das deckt sich mit einer Befragung von Auszubildenden hinsichtlich ihres Fortbildungsinteresses (Buhtz et al. 2020). Das zeigt einerseits den notwendigen Handlungsbedarf für Verbesserungen im Berufsfeld der Pflege und andererseits das latente Interesse für Wissenserwerb im Bereich DAT. Die Pflegefachpersonen konstatieren, durch das positive Erleben des Einsatzes von DAT und des 3‑schrittigen Edukationskonzepts besser für die Anwendung qualifiziert zu sein. Die Pflegefachpersonen machen deutlich, dass sie das Gefühl haben, durch den Edukationsansatz aus Sensibilisierung, Qualifizierung und Erprobung einen ersten Schritt hin zu erhöhter Anwendungskompetenz getan zu haben. Der strukturierte Ansatz gab dem Pflegefachpersonal die Möglichkeit, den Umgang mit DAT in einer bekannten Umgebung zu erlernen und erworbenes Wissen und Fähigkeiten praktisch anzuwenden.

## Stärken und Limitationen

Das gewählte qualitative Studiendesign war geeignet, da es eine erste Hinführung zur Thematik unterstützte und somit erste Eindrücke und Erfahrungen der Pflegefachpersonen erhoben werden konnten. Bei den Fragen des Interviewleitfadens gibt es weiteren Anpassungsbedarf, da nicht alle Fragen als verständlich aufgefasst wurden und die Beantwortung so möglicherweise eingeschränkt war. Durch zu konkret gestellte Fragen des dreigeteilten Interviewleitfadens wurden die Interpretationsspielräume möglicherweise limitiert. Hier wäre ein eher offener Fragenmodus zielführender gewesen. Kritisch ist anzumerken, dass bei diesem Edukationsansatz zu DAT möglicherweise Lernprozesse instrumentalisiert und die Lernenden zu wenig zu einem kontroversen Diskurs über DAT ermutigt werden. Theoretisch könnte angeführt werden, dass sich im Antwortverhalten der Interviewten Ansätze von sozialer Erwünschtheit („confirmation bias“) wiederfinden, da die „lehrende“ und die „interviewende“ Person identisch waren. Dieser Aspekt ist beachtenswert, gerade auch mit Blick auf die Bereitschaft, positive Antworten im Sinne der Projektdurchführung zu generieren. Dazu ist anzumerken, dass der damit zu erzielende Effekt, nämlich in den Augen anderer positiv zu erscheinen, eine in die Zukunft gerichtete Option ist. Die Einmaligkeit der Pilotstudie war jedoch allen Pflegefachpersonen bekannt, sodass von einem persönlichen Benefit durch sozial erwünschtes Antwortverhalten von vornherein nicht auszugehen war. Dennoch war von einer gewissen Sympathie gegenüber der Mentorin auszugehen, da diese den Einsatz 3 Wochen lang intensiv begleitete. Diese Verzerrungspotenziale sind jedoch in einem auf Längerfristigkeit und persönlicher Nähe beruhenden Feldforschungsprojekt nie gänzlich auszuschließen. Ebenfalls werden wichtige Fragen bei einem stark instrumentellen Bildungsansatz ausgeklammert: Wo kommt das relevante Transformationswissen her? Wer hat die Themen und Wissensgebiete für das Lehr‑/Lernsetting ausgesucht, und nach welchen Maßstäben wird bewertet?

Weder das Personal des Wohnbereiches noch die Befragten hatten vorher Kontakt zu DAT und waren daher gänzlich unerfahren. Eine Stärke der Untersuchung ist die mehrwöchige Implementierung auf einem Wohnbereich, wodurch sich der starke Praxisbezug der Untersuchung ergibt. Reale Umstände des Pflegealltags bilden damit den Rahmen. Da die Interviews in geringem zeitlichem Abstand zu der Intervention geführt wurden, lassen sich keine Aussagen zur Nachhaltigkeit gerade mit Blick auf den Einsatz von DAT in der Pflege treffen. Antworten auf diese Frage sind mit Blick auf den Einsatz technischer Hilfsmittel von zentraler Bedeutung und sollten im Rahmen weiterführender Forschungstätigkeiten untersucht werden.

Die Eingrenzung auf 3‑jährig examinierte Pflegefachpersonen stellte sich als zu einschränkend dar, da sich das Personal einer solchen Einrichtung aus weiteren Berufsgruppen (z. B. Pflegehelfern und Pflegehelferinnen) zusammensetzt, die ebenfalls eine besondere Relevanz für die Versorgung haben und von der Nutzung bestimmter DAT nicht ausgeschlossen sein dürfen. Das bedeutet, dass weiterhin Therapieberufe wie Ergotherapeuten- und Therapeutinnen sowie Beschäftigungstherapeuten- und Therapeutinnen perspektivisch miteinzubeziehen sind.

## Schlussfolgerung

Der strukturierte Ansatz aus Sensibilisierung, Schulung und Erprobung wurde von den Befragten als sinnvoll erachtet. Das Vorgehen ermöglichte eine Form der schrittweisen Wissens- und Kompetenzvermittlung und die Erweiterung und Neubetrachtung bereits vorhandener Wissensbestände. Nach der Vermittlung von theoretischem Wissen, der Anwendungsschulung und Praxiserprobung trug der Ansatz dazu bei, Passgenauigkeit von DAT auf im Vorhinein durch Pflegevisiten identifizierte Pflegeprobleme herzustellen. Aufgrund der engen zeitlichen Ressourcen sollen weitere Berufsgruppen in den Prozess miteinbezogen werden. Diese können den Einsatz der Therapie- und Kommunikationsroboter mit den Bewohnern und Bewohnerinnen übernehmen.

Die interviewten Pflegefachpersonen sehen die wertschätzende Vermittlung auf Augenhöhe bei der Einführung von DAT im Rahmen dieses Edukationsansatzes als wichtig an.

Perspektivisch ist es wichtig, die benötigten Kompetenzen und Qualifikationen für Pflegefachpersonen als potenzielle Multiplikatoren und Multiplikatorinnen zu klären, um andere Pflegefachpersonen im Umgang mit DAT zu qualifizieren. Die langzeitstationäre Einrichtung plant aufgrund der wahrgenommenen positiven Auswirkungen auf Bewohner und Bewohnerinnen (interne Evidenz), einen therapeutischen Roboter PARO zu beschaffen, was auf eine erfolgreiche Umsetzung des Ansatzes schließen lässt. Da der Forschungsschwerpunkt dieser Studie auf den Pflegefachpersonen liegt, können keine Aussagen zu Akzeptanz oder Ablehnung der Bewohner und Bewohnerinnen bezüglich der DAT getroffen werden. Weiterführend bleibt zu untersuchen, welche DAT in einer versorgenden Praxis in welchem Kontext angewandt werden, und inwieweit sich die Ergebnisse des Edukationsansatzes auf alle DAT gleichermaßen übertragen lassen. Ebenfalls gilt es in weiterführenden Arbeiten, mehr darüber zu erfahren, wie sich DAT auf vorher festgelegte Pflegeziele konkret auswirken.

## Supplementary Information





