# Supplementary material for: Identification of measures conducive to learning for the introduction of digital and assistive technologies (DAT) in processes of nursing care: a qualitative study
Source: HeilberufeScience. 2022 Jun 14;13(3-4):152–61. [Article in German] doi: 10.1007/s16024-022-00372-4 (PMC9195397; doi:10.1007/s16024-022-00372-4)
Supplement: Supplementary file 1 [file 16024_2022_372_MOESM1_ESM.docx]

**Identifikation von lernfördernden Maßnahmen zur Einführung von digital, assistiven Technologien (DAT) in Prozesse der pflegerischen Versorgung: Eine qualitative Studie**

Zusätzliches Onlinematerial 1 -

PARO

…ist ein interaktiver, therapeutischer Tierroboter (TTR), der eine Babysattelrobbe imitiert. Unter dem weißen Kunstfell befinden sich Sensoren, die eine Interaktion mit Dritten ermöglichen (Robinson, MacDonald, Kerse & Broadbent, 2013). PARO ist im Kopfbereich, an den Vorderflossen und im Bereich der Schwanzflosse beweglich und reagiert auf Berührungen. Das robotische System kann Vorlieben der Nutzerinnen und Nutzer erlernen und auf negative Einflüsse reagieren, wenn es beispielweise geschlagen wird (Pu, Moyle, Jones & Todorovic, 2020). Vorliegende Arbeiten zum Einsatz von PARO als Intervention im Umgang mit dementiell erkrankten Menschen zeigten positive Effekte z.B. bei Depression, Schmerzen, Agitation, psychosozialer Gesundheit, chronischen Schmerzen und der Stimmung (Jøranson, Pedersen, Rokstad & Ihlebæk, 2015; Pu, Moyle & Jones, 2020; Pu et al. 2020; Robinson et al., 2013).

PLEO

…wird als emotionaler und therapeutischer Roboter beschrieben. Seine Ursprünge hat er allerdings in der Kreativwirtschaft (Kinderspielzeug). Das robotische System imitiert einen pflanzenfressenden Dinosaurier und fördert Fürsorge- und Zuwendungsverhalten der Nutzerinnen und Nutzer. Zudem „durchlebt“ es verschiedene Entwicklungsstadien, die durch die Häufigkeit der Interaktion beeinflussbar sind. Das System verfügt über Berührungs-, Bewegungs- und optische Sensoren und kann spezielle Objekte mit dem Maul ergreifen (Baisch et al., 2018; Dimas et al., 2010; Domínguez-Rué & Nierling, 2016; Fernaeus, Håkansson, Jacobsson & Ljungblad, 2010).

Passive Exoskelette

Exoskelette können verschiedene Personengruppen bei körperlicher Anstrengung entlasten (Klein et al., 2018). Es wird zwischen aktiven und passiven Exoskeletten unterschieden. Aktive Exoskelette verfügen neben mechanischen Elementen über weitere aktive Antriebskomponenten, die Kräfte reduzieren und zur Lastminderung beitragen. Passive Exoskelette hingegen verfügen nicht über aktive Antriebskräfte und unterstützen den tragenden Körper lediglich mittels mechanischer Komponenten wie Gurtsystemen und Schienen. Das bedeutet, dass entstehende Belastungen von der Stützstruktur aufgenommen, in Bewegungsenergie überführt und in den Boden abgeleitet werden. Ein passives Exoskelett nutzt keine externe Energiezufuhr, lediglich Material, um Gewicht zu verteilen, Stöße abzudämpfen oder Energie zu speichern und wieder frei zu geben (Bosch et al., 2016).

Die folgenden Exoskelette gehören der Gruppe der passiven Exoskelette an:

Das Exoskelett von „Hunic“ unterstützt die Ergonomie der Körperhaltung. Durch eine Schiene entlang der Wirbelsäule wird eine unphysiologische Beugung der Wirbelsäule verhindert (Hunic.com, 2020). Das bedeutet, es hilft Bewegungsabläufe anzupassen und beispielsweise auf die Wirbelsäule einwirkende Belastungen zu minimieren.

Das Exoskelett „Laevo“ verfolgt einen anderen Ansatz. Durch ein Schienensystem und spezifische Mechanik wird auf die Lendenwirbelsäule einwirkende Gewichtkraft auf die unteren Extremitäten und den Körperstamm um- und abgeleitet (Bosch et al., 2016).

Telepräsenzsystem Double

Ein Telepräsenzsystem ermöglicht den Nutzerinnen und Nutzer virtuelle Präsenz bei physischer Abwesenheit. Kommunikation wird über eine audiovisuelle Benutzeroberfläche möglich (Klein et al., 2018). Im Gegensatz zu herkömmlichen videobasierten Kommunikationssystemen kann ein Telepräsenzsystem über unterschiedliche Endgeräte (z.B. Smartphone, Tablet, Computer) angewählt und manövriert werden. Das durch uns verwendete Telepräsenzsystem ist das „Double 3“ (Doublerobotics.com, 2020).

Literaturverzeichnis:

Baisch, S., Kolling, T., Rühl, S., Klein, B., Pantel, J., Oswald, F., & Knopf, M. (2018). Emotionale Roboter im Pflegekontext: Empirische Analyse des bisherigen Einsatzes und der Wirkungen von Paro und Pleo. In: Zeitschrift fur Gerontologie und Geriatrie, 51(1), 16–24. https://doi.org/10.1007/s00391-017-1346-8

Bosch, T., van Eck, J., Knitel, K., & de Looze, M. (2016). The effects of a passive exoskeleton on muscle activity, discomfort and endurance time in forward bending work. In: Applied Ergonomics, 54, 212–217. https://doi.org/10.1016/j.apergo.2015.12.003

Dimas, J., Leite, I., Pereira, A., Cuba, P., Prada, R., & Paiva, A. (2010). Pervasive Pleo: Long-term Attachment with Artificial Pets. Workshop on playful experiences in Mobile HCI. Abgerufen von http://dl.lirec.org/papers/Dimas.et.al-ACM_MobileHCI2010_workshop.pdf Abgerufen am: 10.01.2021

Domínguez-Rué, E., & Nierling, L. (2016). Ageing and technology : perspectives from the social sciences. In: Zeitschrift fur Gerontologie und Geriatrie, Bd. 29, S. 246–256. Abgerufen von https://www.degruyter.com/doc/cover/9783839429570.jpg%0Ahttps://www.degruyter.com/openurl?genre=book&isbn=9783839429570%0Ahttps://search.ebscohost.com/login.aspx?direct=true&scope=site&db=nlebk&db=nlabk&AN=1164193%0Ahttps://www.jstor.org/stable/10.2307/j.

Doublerobotics.com. (2020). Double 3 - Overview. Abgerufen 27. August 2020, von https://www.doublerobotics.com/double3.html

Fernaeus, Y., Håkansson, M., Jacobsson, M., & Ljungblad, S. (2010). How do you play with a robotic toy animal? Narcís Parés und Miquel Oliver (Hg.): Proceedings of the 9th International Conference on Interaction Design and Children - IDC '10 the 9th International Conference. Barcelona, Spain, 09.06.2010 - 12.06.2010. New York, New York, USA: ACM Press, S. 39.“, 39. https://doi.org/10.1145/1810543.1810549

Hunic.com. (2020). Hunic- Exoskelett für die Industrie. Abgerufen 27. August 2020, von https://hunic.com/

Jøranson, N., Pedersen, I., Rokstad, A. M. M., & Ihlebæk, C. (2015). Effects on Symptoms of Agitation and Depression in Persons With Dementia Participating in Robot-Assisted Activity: A Cluster-Randomized Controlled Trial. Journal of the American Medical Directors Association, 16(10), 867–873. https://doi.org/10.1016/j.jamda.2015.05.002

Klein, B., Graf, B., Schlömer, F., Roßberg, H., Röhricht, K., & Baumgarten, S. (2018). Robotik in der Gesundheitswirtschaft Einsatzfelder und Potentziale (Stiftung Münch, Hrsg.). Abgerufen von https://www.medhochzwei-verlag.de/Shop/ProduktDetail/robotik-in-der-gesundheitswirtschaft-buch-978-3-86216-388-5

Pu, L., Moyle, W., & Jones, C. (2020). How people with dementia perceive a therapeutic robot called PARO in relation to their pain and mood: A qualitative study. Journal of Clinical Nursing, 29(3–4), 437–446. https://doi.org/10.1111/jocn.15104

Pu, L., Moyle, W., Jones, C., & Todorovic, M. (2020). The Effect of Using PARO for People Living With Dementia and Chronic Pain: A Pilot Randomized Controlled Trial. Journal of the American Medical Directors Association, 1–7. https://doi.org/10.1016/j.jamda.2020.01.014

Robinson, H., MacDonald, B., Kerse, N., & Broadbent, E. (2013). The Psychosocial Effects of a Companion Robot: A Randomized Controlled Trial. Journal of the American Medical Directors Association, 14(9), 661–667. https://doi.org/10.1016/j.jamda.2013.02.007

Valentí Soler, M., Agüera-Ortiz, L., Olazarán Rodríguez, J., Mendoza Rebolledo, C., Pérez Muñoz, A., Rodríguez Pérez, I., … Martínez Martín, P. (2015). Social robots in advanced dementia. Frontiers in Aging Neuroscience, 7(JUN). https://doi.org/10.3389/fnagi.2015.00133
