# Supplementary material for: Identification of measures conducive to learning for the introduction of digital and assistive technologies (DAT) in processes of nursing care: a qualitative study
Source: HeilberufeScience. 2022 Jun 14;13(3-4):152–61. [Article in German] doi: 10.1007/s16024-022-00372-4 (PMC9195397; doi:10.1007/s16024-022-00372-4)
Supplement: Supplementary file 2 [file 16024_2022_372_MOESM2_ESM.docx]

**Online-Material 2: Interviewleitfaden**

**Einstiegsfrage:**

- Wie haben Sie die Nutzung der Technologien hier in Ihrer Einrichtung empfunden und erlebt?

**Fragenkomplex - Kategorien**

A. Allgemeine Schilderung zu dem Einsatz von DAT

- Können Sie Ihre Gedanken über den erlebten Einsatz von den assistiven Technologien und Robotern im Rahmen der Versorgung Ihrer Bewohner*innen schildern?
- Wo sehen Sie in der täglichen Arbeit Probleme?

B. Angewandte Methodenwahl und allgemeine Fragen zu den einzelnen Schritten des Schulungsansatzes

- Empfanden Sie die Schulung als hilfreich, um auf den Einsatz assistiver Technologien und Roboter im Rahmen ihrer pflegerischen Arbeit in Zukunft besser vorbereitet zu sein?
- Wie fanden Sie den Aufbau der Schulung, d.h. die Schritte aus Sensibilisierung, Qualifizierung und Praxiserprobung?
- Ist ihre Bereitschaft nach der Schulung erhöht Assistenztechnologien in der Pflege einzusetzen?
- Wie müssten Weiter-und Fortbildungen zum Bereich Assistenztechnologien Ihrer Meinung nach aussehen? Hätten Sie Vorschläge unser Vorgehen zu verbessern?

C. Spezifizierung und Vertiefung / Reflexion der selbst gewählten Szenarien anhand der Bereiche der Pflegebedürftigkeit

- Können Sie Situationen beschreiben, in denen Sie den Einsatz als hilfreich empfunden haben? (Weshalb?)
- Können Sie Situation beschreiben, in denen Sie den Einsatz als schwierig empfanden? (Weshalb?)

**Schluss:**

- Haben Sie noch Fragen? Gibt es aus Ihrer Sicht noch etwas, dass Sie sagen/ergänzen möchten? Sind Dinge offengeblieben?
- Haben Sie sonst noch Wünsche oder Anregungen?
